# RECRUITMENT SUCCESSES AND CHALLENGES OF EMPLOYING IN-HOME SENSOR TECHNOLOGY WITH OLDER AFRICAN AMERICAN ADULTS

**DOI:** 10.1093/geroni/igad104.1459

**Published:** 2023-12-21

**Authors:** Shayla Calloway, Journie Raulston, Lisa Barnes

**Affiliations:** Rush University Medical Center, Chicago, Illinois, United States; Rush University, Chicago, Illinois, United States; Rush University Medical Center, Chicago, Illinois, United States

## Abstract

Older African Americans are under-represented in clinical research studies of aging. Many previous studies have shown results of in-home monitoring technologies to collect objective data for aging in place but few studies include minoritized populations. The CART platform uses home-based sensor technology to unobtrusively assess activity in the home and was embedded in homes of participants from the Minority Aging Research Study (MARS), an ongoing longitudinal study of cognitive decline in older African Americans. This presentation will highlight efforts to engage, recruit, and retain older African American participants in Chicago, with a particular focus on challenges and lessons learned. Recruitment consisted of presentations and mailings to potentially eligible MARS participants based on pre-specified inclusion criteria. Of 153 participants who met initial eligibility criteria, 36 refused before consent, 34 were ineligible after screening, and we enrolled 76 non-Hispanic African Americans participants (80% women; mean age = 80.4 (6.3) years); mean education = 16 (2.8) years; 78% without cognitive impairment). Enrolled participants were slightly younger, more likely to be female, and had higher education. There was no difference in cognitive impairment between those who enrolled compared to those who were ineligible after screening. Since enrollment, 11 have withdrawn and 7 are no longer active. The most common challenges include privacy concerns, broadband internet issues, emerging medical conditions, or changes in residency or resident occupancy. These results demonstrate that older African Americans participating in an ongoing longitudinal study can be recruited and retained in an ancillary study that involves home-based sensor technology.

